# Clinical effectiveness and cost-effectiveness of pegvisomant for the treatment of acromegaly: a systematic review and economic evaluation

**DOI:** 10.1186/1472-6823-9-20

**Published:** 2009-10-08

**Authors:** David J Moore, Yaser Adi, Martin J Connock, Sue Bayliss

**Affiliations:** 1 Public Health, Epidemiology and Biostatistics, School of Health and Population Sciences, University of Birmingham, Birmingham, B15 2TT, UK; 2 Health Sciences Research Institute, Warwick Medical School, University of Warwick, Coventry, CV4 7AL, UK

## Abstract

**Background:**

Acromegaly, an orphan disease usually caused by a benign pituitary tumour, is characterised by hyper-secretion of growth hormone (GH) and insulin-like growth factor I (IGF-1). It is associated with reduced life expectancy, cardiovascular problems, a variety of insidiously progressing detrimental symptoms and metabolic malfunction. Treatments include surgery, radiotherapy and pharmacotherapy. Pegvisomant (PEG) is a genetically engineered GH analogue licensed as a third or fourth line option when other treatments have failed to normalise IGF-1 levels.

**Methods:**

Evidence about effectiveness and cost-effectiveness of PEG was systematically reviewed. Data were extracted from published studies and used for a narrative synthesis of evidence. A decision analytical economic model was identified and modified to assess the cost-effectiveness of PEG.

**Results:**

One RCT and 17 non-randomised studies were reviewed for effectiveness. PEG substantially reduced and rapidly normalised IGF-1 levels in the majority of patients, approximately doubled GH levels, and improved some of the signs and symptoms of the disease. Tumour size was unaffected at least in the short term. PEG had a generally safe adverse event profile but a few patients were withdrawn from treatment because of raised liver enzymes. An economic model was identified and adapted to estimate the lower limit for the cost-effectiveness of PEG treatment versus standard care. Over a 20 year time horizon the incremental cost-effectiveness ratio was £81,000/QALY and £212,000/LYG. To reduce this to £30K/QALY would require a reduction in drug cost by about one third.

**Conclusion:**

PEG is highly effective for improving patients' IGF-1 level. Signs and symptoms of disease improve but evidence is lacking about long term effects on improved signs and symptoms of disease, quality of life, patient compliance and safety. Economic evaluation indicated that if current standards (UK) for determining cost-effectiveness of therapies were to be applied to PEG it would be considered not to represent good value for money.

## Background

Acromegaly is a rare endocrine disorder resulting from excessive secretion of growth hormone (GH) [1,2]. The underlying cause in more than 90% of patients is a benign adenoma of the GH-secreting cells of the anterior pituitary. Very rarely acromegaly is due to hypothalamic over secretion of growth hormone releasing hormone (GHRH) or to extra-pituitary tumours that secrete GH or GHRH.

GH promotes insulin-like growth factor-1 (IGF-1) secretion. Prolonged exposure to elevated endogenous levels of GH and/or IGF-1 in acromegaly results in excessive somatic growth and metabolic dysfunction leading to both direct and indirect tissue damage, secondary systemic illness and reduced life expectancy. Extended discussion of the systemic complications can be found in Colao et al 2004 [3], Melmed 2006 [1] and Chanson and Salenave[2]

The insidious development of symptoms and their variety contribute to delayed diagnosis of about 8 years from onset of first symptoms [4]. Biochemical diagnosis is made by immunoassay of GH and of IGF-1 in blood [1].

Traditionally excess secretion of GH has been targeted by treatment strategies to reduce secretion; employing surgery, radiotherapy, dopamine agonists (DOPAs), and/or somatostatin agonists (SSAs) alone or as adjuvant to surgery. Recently Pegvisomant (Somavert^®^; Pfizer) was developed with the aim of blocking the action of circulating GH. Pegvisomant (PEG) is a genetically engineered analogue of human GH that can compete with endogenous GH for GH receptors while failing to activate the receptor. As the effectiveness of PEG depends on competition with GH the required dose is influenced by endogenous GH levels which depend on the size, activity and type of adenoma. PEG is administered daily by subcutaneous injection. In Europe, PEG is licensed for patients who have had an inadequate response to surgery and/or radiation and in whom an appropriate medical treatment with somatostatin analogues did not normalise IGF-1 concentrations or was not tolerated[5].

Here we present a systematic review of the evidence about the clinical effectiveness of PEG and an economic analysis to estimate the cost-effectiveness of PEG treatment relative to standard care.

## Methods

The review was conducted according to a predefined protocol (available on request).

### Search strategy clinical effectiveness

The following bibliographic databases and other sources were searched for studies of clinical effectiveness: (i) Bibliographic databases: Cochrane Library (Wiley), MEDLINE (Ovid), MEDLINE (Ovid) In-Process, EMBASE (Ovid), CINAHL (EBSCO). (ii) Sources of information on ongoing and unpublished research (including the National Research Register and ClinicalTrials). (iii) Sources of Abstracts and Proceedings (ZETOC, ENDO 2006 Endocrine Society's 88^th ^annual meeting). (iv) Citations of relevant studies. (v) Experts in the field were contacted to check that no published or unpublished studies had been missed. (vi) Studies listed in systematic and other reviews. The following bibliographic databases were searched for economic studies: MEDLINE(Ovid); EMBASE (Ovid); Cochrane Library (Wiley); NHS EED, OHE HEED. Electronic databases were searched up to March 2007. No language or date restrictions were applied. Full details are in Additional File 1.

### Inclusion and exclusion criteria for clinical studies

No systematic reviews were identified. Primary studies of effectiveness were selected according the following criteria: *Study design*: RCTs, quasi-randomised clinical trials, comparative non-randomised studies, or case series if at least 10 patients were included. *Population*: Patients diagnosed with acromegaly. *Intervention*: Treatment with PEG. *Comparator(s): *any other or no treatment. *Outcomes*: Any clinically relevant outcomes, changes in IGF-1 levels and GH levels. Economic studies were selected using the following criteria: *Study design*: Cost-effectiveness, cost-utility and cost-benefit studies. Health economic reviews were also included. *Population*: People with acromegaly. *Intervention*: PEG. *Comparator*: Any alternative treatment. *Outcomes*: Quality of life, costs or incremental cost-effectiveness ratio. Conference and symposium abstracts were noted and used to check for studies published as full papers.

The quality of included studies was assessed using standard check lists [6], and appropriate data were extracted from included studies by one reviewer and checked by a second. Application of inclusion criteria, quality assessment and data extraction were undertaken by one reviewer and checked by a second. Disagreements were resolved by consensus.

Heterogeneity of clinical studies precluded meta-analysis and clinical effectiveness was reviewed by narrative synthesis. Studies that were multiply published were checked and the most appropriate trial data extracted. Studies that reported health-related quality of life (QoL) results for patients with acromegaly but did not satisfy the inclusion criteria were noted and if judged relevant were used to inform the economic analysis.

## Results

### Number and type of studies identified

The electronic search yielded 319 citations (see Additional File 2). After removal of duplicates and irrelevant citations on the basis of title and/or abstract the full texts of 32 citations were obtained for further scrutiny. On application of inclusion criteria 14 publications were excluded (see Additional File 2 for reasons for exclusion). The main characteristics of the eighteen included publications are summarised in Additional File 3 (further details are provided in Additional File 4). The studies comprised one multicentre RCT (Trainer 2000 [7]) of 112 patients randomised to placebo or three different doses of PEG for 12 weeks; an open label extension of this RCT (Van der Lely 2001 [8]) with additional patients (total = 160) and altered dose regimen; two publications describing analyses of patient subgroups from the RCT [9,10]; a retrospective case series (n = 142) aimed at monitoring safety of PEG treatment (Biering 2006 [11]); and 13 before versus after PEG treatment comparisons [12-24], one of which [12] was conducted retrospectively. Three of these publications (Paisley 2006; [13] Parkinson 2004 and Parkinson 2003 [14,15]) included a comparison with matched healthy subjects in a cross sectional (i.e. single-time measure) design. In several instances the same patients were involved in more than one of the above studies.

### Populations recruited

PEG is licensed for patients with suboptimal response to other treatments or who are intolerant of medical treatment required to normalise GH and IGF-1 levels. The RCT (Trainer 2000 [7]) was conducted prior to licensing. The status of patients relative to the licensed indication is not clear. One study, Colao 2006 [16], did recruit only patients that had not satisfactorily responded to other treatments.

### Dose regimens and duration of treatment

In most studies a large loading dose (40 to 80 mg) of PEG was administered on day one. After the loading dose in the RCT doses of 10, 15 or 20 mg/day were given for 12 weeks. In most other studies, after loading, 10 mg/day was administered but adjusted at timed intervals until serum IGF-1 levels had declined to within normal range or a maximum dose (e.g. 30 or 40 mg/day) was reached. The dose regimen was halted, suspended or reduced if serum liver enzymes rose to levels giving clinical concern. Three studies [17-19] employed distinctly different dose regimens from other studies. In Jehle 2005 [17] (n = 10), after achieving IGF-1 normalisation, the interval between dosing was first doubled (dose every other day) and then, if IGF-1 normalisation was retained, doubled again. If, after dose-frequency change, IGF-1 reverted to abnormal levels then dose frequency was returned to the previously successful frequency. In Jorgensen 2005 [19] (n = 11) PEG was compared to PEG combined with long-acting SSA. In Feenstra 2005 [18] PEG was administered weekly rather than daily but was adjunct to monthly administration of long-acting SSA treatment, and PEG dose was increased until IGF-1 normalisation was achieved.

Follow-up of patients was short term in most studies. Duration of study treatment period varied from as little as 12 weeks in many studies to 12 months in a few, or in the more extended studies up to 2 years for a few patients [8,9,17,20,21] (Additional File 3).

### Outcomes reported

Signs and symptoms of disease were monitored in the RCT and three other studies [7,16,17,21] using patient questionnaires. Serum IGF-1 levels were almost universally reported. GH levels were reported in the RCT and its extension [7,8] and a few small studies [9,16,19]. Several studies focussed on risk factors for cardiovascular disease [13,16,20,22], and/or for diabetes [17,19,20,23]. Two studies focussed on markers of bone metabolism [10,15]. Side effects and transaminases levels were commonly, but not universally, reported. None of the studies assessed quality of life outcomes.

### Risk of bias in included studies

Withdrawals from treatment in the RCT were fully described and trial arms were balanced at baseline; the publication provided few details of randomisation, allocation concealment or blinding procedures. The greatest risks for bias amongst the remaining studies (Table 1) arose from a lack of clear information about the sampling frame from which study participants had been selected and a lack of description of the selection methods employed. The rarity of acromegaly may have dictated the use of convenience samples in most studies but this was not explicitly reported.

**Table 1:** Assessment of risks to bias in non-randomised studies

**Study**	**Were eligibility criteria explicit?**	**Was sample source/selection described?**	**Were patients assembled at same time?**	**Was a method of diagnosis stated?‡**	**Were clinical details described?**	**Was individual patient data reported?**	**Was outcome assessment blinded?**	**Was blinding method adequately described?**	**Was follow up time stated?Φ**	**Were withdrawals stated?**	**Were reasons for withdrawal stated?**
Barkan 2005[24]	**Y**	**N**	**CT**	**N**	**Y**	**N**	**N**	**NA**	**Y**	**Y**	**Y**

Jorgensen 2005[19]	**Y**	**Y**	**CT**	**N**	**Y**	**N**	**N**	**NA**	**Y**	**Y**	**Y**

Feenstra 2005[18]	**Y**	**N**	**CT**	**N**	**Y**	**N**	**N**	**NA**	**Y**	**N**	**NA**

Van der Lely 2001[8]	**Y**	**N**	**N**	**N**	**Y**	**N**	**N**	**NA**	**Y**	**Y**	**Y**

Sesmilo 2002[9]	**Y**	**N**	**CT**	**Y**	**Y**	**N**	**N**	**NA**	**Y**	**Y**	**Y**

Fairfield 2002[10]	**N**	**N**	**CT**	**N**	**Y**	**N**	**N**	**NA**	**Y**	**N**	**NA**

Parkinson 2002[22]	**N**	**N**	**CT**	**N**	**Y**	**Y†**	**N**	**NA**	**N**	**N**	**NA**

Parkinson 2003a[15]	**Y**	**N**	**CT**	**N**	**Y**	**N**	**N**	**NA**	**N**	**N**	**NA**

Parkinson 2003b[23]	**N**	**N**	**CT**	**N**	**Y**	**Y†**	**N**	**NA**	**Y**	**N**	**NA**

Parkinson 2004[14]	**N**	**N**	**CT**	**N**	**N**	**Y†**	**N**	**NA**	**N**	**N**	**NA**

Jehle 2005[17]	**N**	**N**	**CT**	**N**	**Y**	**Y**	**N**	**NA**	**Y**	**Y**	**Y**

Paisley 2006[13]	**N**	**N**	**CT**	**N**	**Y**	**Y**	**N**	**NA**	**N**	**N**	**N**

Biering 2006[11]	**CT**	**Y**	**NA**	**N**	**N**	**Y^a^**	**N**	**NA**	**N**	**Y**	**Y**

Colao 2006[16]	**Y**	**Y**	**Y**	**Y**	**Y**	**Y**	**N**	**NA**	**Y**	**Y**	**Y**

Pivonello 2007[20]	**Y**	**N**	**CT**	**Y**	**Y**	**Y**	**Y**	**Y**	**Y**	**Y**	**N**

Schreiber 2007[21]	**N**	**Y**	**N**	**N**	**Y**	**N**	**N**	**NA**	**Y**	**Y**	**N**‡‡

Parkinson 2007[12]	**Y**	**N**	**CT**	**N**	**Y**	**Y**	**Y**	**N**	**N**	**N**	**NA**

^a ^for subgroup of patients. ‡ in most studies this was implicit ("patients with established diagnosis") rather than explicit. † in graphs. Φ where patient follow up varied but group value only provided N is entered. ‡‡ for adverse events only.

Y = yes; N = no; CT = can't tell; NA = not applicable. Assessment used recommendations in the CRD handbook 2'edition [6]

### Clinical effectiveness: signs and symptoms of acromegaly

**Figure 1:**
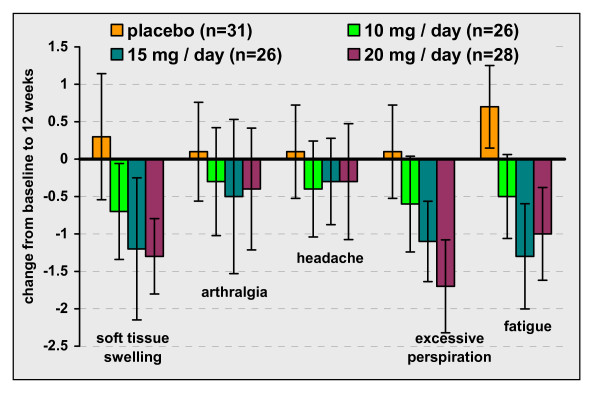
**Change in signs and symptoms of acromegaly reported in the RCT**. Mean and 95% confidence intervals of change at 12 weeks relative to baseline. Raw data taken from Trainer 2000 [7].

The RCT [7] elicited patient information on signs and symptoms using a questionnaire rating scale of 0 (no symptoms) to 8 (severe, incapacitating) for each of five symptom categories: soft tissue swelling, headache, joint pain, excessive sweating, fatigue (Figure 1). At 12 weeks statistically significant (p ≤ 0.05) improvements from baseline were noted for the two high dose groups for soft tissue swelling and excessive perspiration, and for all three PEG groups for fatigue. No statistically significant changes occurred for the placebo group except for fatigue which worsened.

The small uncontrolled study of Jehle [17] (N = 10) noted a trend for improvement over a mean treatment period of 15.3 (± 4.6) months. Colao [16] reported a favourable trend toward improvement from baseline for 10 patients; none of the changes in the individual symptom category scores reached statistical significance. Schreiber [21] reported statistically significant improvements (6 months vs. baseline score) for soft tissue swelling, headache, joint pain, general physical condition, and for total score. In this study 62 patients (of 229) completed the questionnaire at baseline and 56 at 6 months into treatment. The results may be susceptible to sampling bias. Both the RCT and Jehle study reported statistically significant reductions in finger-ring size that were attributed to PEG therapy.

### Clinical effectiveness: tumour volume

In the RCT PEG treatment did not alter the group mean tumour volumes relative to baseline and no individual patient exhibited a clinically significant increase in tumour volume [7]. In the RCT extension [8] (N = 131) 160 MRI image pairs were collected, one image at baseline and another at an average of 11.5 months into treatment. No statistically significant change from baseline was observed in mean tumour volume. At baseline mean tumour volume was 2.41 ml (95% CI: 1.8 to 3.0) and after treatment was 2.37 ml (95% CI: 1.8 to 3.0). The mean of individual change from baseline was - 0.033 ml (95% CI: - 0.15 to + 0.08; p = 0.353 versus zero change). Two patients had progressive tumour growth requiring treatment, the authors could attribute no cause, and there was no relationship between duration of treatment and change in tumour size. Colao [16] reported a baseline mean tumour size of 1.23 ml (95% CI: 0.55 to 1.91) for 14 patients; after treatment mean volume was 1.20 ml (95% CI; 0.46 to 1.95); the mean change in volume was - 0.026 ml (95% CI; - 0.21 to + 1.56). Jehle [17] observed small clinically insignificant increases in tumour size in 2 of 10 patients (duration of treatment 12 to 20 weeks). Dual-therapy (PEG + SSA) studies [18,19] reported similar results (statistically non-significant).

### Clinical effectiveness: achievement of normal IGF-1 levels

**Figure 2:**
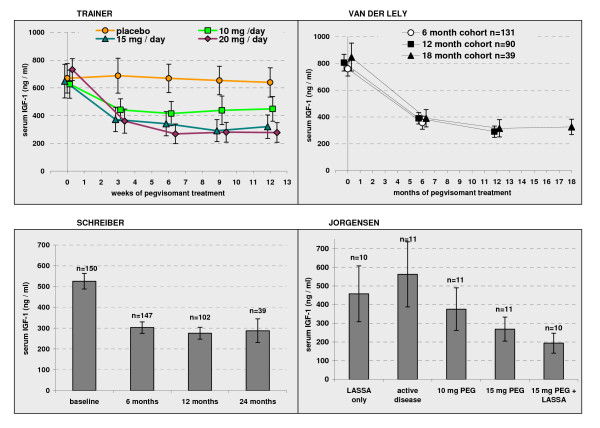
**Decline in IGF-1 levels with PEG treatment**. Baseline and follow up mean values with 95% confidence intervals reported in randomised [7] and non-randomised studies. The duration of treatment in the 5 phases of the Jorgensen study [19] was 2 to 4 weeks, 8 weeks, 6 weeks, 6 weeks and 12 weeks. Dose of PEG in the RCT extension [8] and in Schreiber [21] varied according to patient response. Figure compiled from published data [7,8,19,21] with the addition of 95% CI.

In the RCT [7] IGF-1 normalisation was the primary outcome. Baseline IGF-1 levels were at least 1.3 times above the top of the normal range. Statistically significant reductions in IGF-1 occurred after treatment in all three PEG groups but not the placebo group (Figure 2); at all time intervals after baseline statistically significant differences were observed for each PEG regimen versus placebo. A distinct dose response relationship was evident with higher doses more effective than 10 mg PEG/day. At 12 weeks the proportion of patients with normalised IGF-1 levels was 10%, 38%, 75% and 82% in placebo, 10 mg, 15 mg, and 20 mg PEG groups respectively. In the RCT extension [8] PEG dose was titrated to achieve normal range IGF-1 with a maximum allowed dose of 40 mg/day. Figure 2 shows the reported IGF-1 levels. In the cohort treated for 12 months (n = 90) 97% had normalised IGF-1 levels.

Schreiber [21] collected IGF-1 data for 157 of 229 patients at baseline and for 147, 102, and 39 patients after 6, 12 and 24 months (Figure 2). Mean group values were similar to those in the RCT extension [8]. At baseline 11% had normal range IGF-1 and at 6, 12 and 24 months of treatment 64%, 71%, and 76% were in normal range. These percentages are distinctly lower than the 97% reported in the RCT extension. Schreiber et al suggested this may be due to better patient compliance and superior monitoring for dose adjustment in a clinical trial compared to the real world clinical practice reflected in their study. Colao [16] reported individual IGF-1 levels for 16 patients that fitted the licensed indication for PEG. Of 14 patients evaluated at 12 months eight (57%) reduced IGF-1 to within normal range and three more to within 1 to 1.3 times normal range.

Feenstra 2005 [18] and Jorgensen 2005 [19] combined PEG with SSA therapy, whilst Jehle 2005 [17] attempted reduction of dose frequency (Figure 2). Because daily PEG is very expensive these strategies might reduce the overall cost of maintaining IGF-1 within normal range. In Feenstra [18] at 18 weeks IGF-1 was normalised in 21/26 (81%) patients, and at 42 weeks in 95% (18/19 evaluated); the median weekly PEG dose to achieve normalisation in those normalised was 60 mg/week. The Jorgensen study [19] comprised 5 study phases: therapy with SSA, withdrawal from SSA for 2 months, PEG at 10 mg/day (6 weeks), PEG at 15 mg/day (6 weeks), and finally 12 weeks of 15 mg/day PEG combined with 30 mg long acting SSA every 2 to 4 weeks. IGF-1 was measured at the end of each study phase. The final combined therapy reduced IGF-1 to lower levels than single therapy with either LASSA or PEG but the difference just failed to reach statistical significance. The lack of a true control for each phase of study and problems of treatment carryover complicate interpretation of these results. On combined therapy 9 of 10 patients achieved normal IGF-1 levels. Jehle 2005 [17] investigated 10 patients who had failed to normalise IGF-1 with DOPAs or SSAs. Mean PEG treatment was for 15.3 months; all patients normalised IGF-1 and 5 were able to reduce frequency of dose administration to less than daily while retaining a normal IGF-1.

### Clinical effectiveness: effect on GH levels

**Figure 3:**
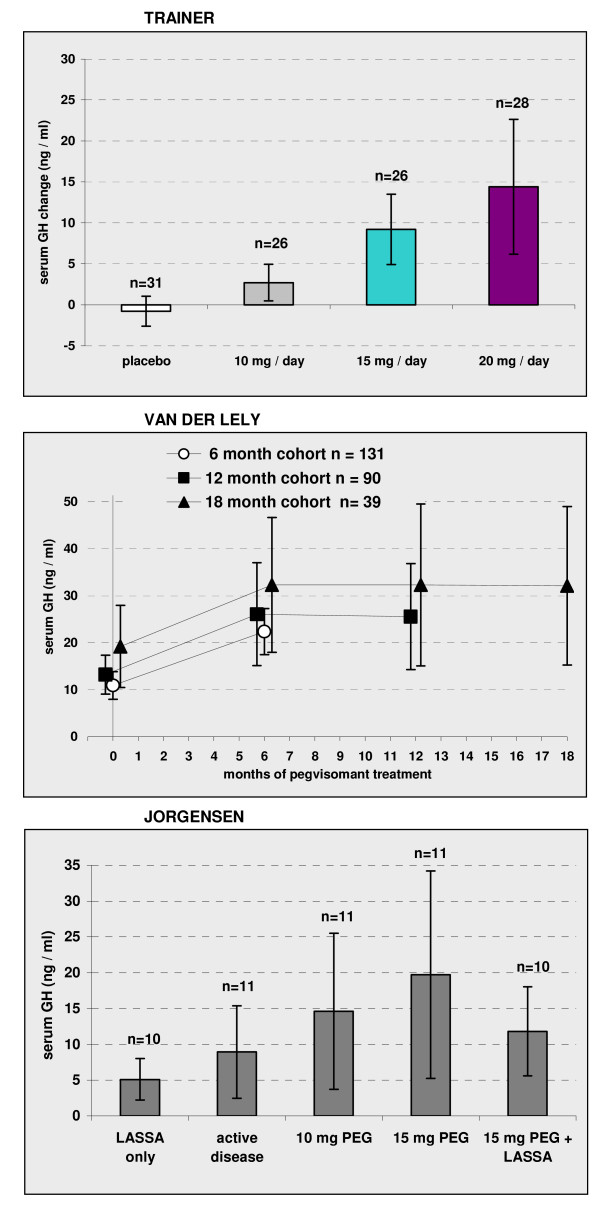
**Increase in GH levels with PEG treatment**. Baseline and follow up mean values with 95% confidence intervals reported in randomised [7] and non-randomised studies. The duration of treatment in the 5 phases of the Jorgensen study [19] was 2 to 4 weeks, 8 weeks, 6 weeks, 6 weeks and 12 weeks. Dose of PEG in the RCT extension [8] varied according to patient response. Figure compiled from published data [7,8,19] with the addition of 95% CI.

In the RCT PEG treatment substantially increased serum GH levels by up to 15 ng/ml above baseline levels (of ~8 ng/ml) and the increase from baseline reached statistical significance for all three dose regimens. However, for patients receiving placebo the change was small and not statistically significant (Figure 3). A dose response relationship was evident with higher dose inducing greater increase. In the RCT extension [8] for the cohort treated for 6 months with PEG the mean GH level was substantially elevated to double that at baseline (Figure 3).

Colao [16] reported individual GH levels for 16 patients. The mean baseline GH ranged from 3.4 to 74.8ng/ml (mean 23ng/ml; 95% CI: 10.9 to 35.0). After treatment, discounting one patient who failed to inject PEG, the range was 6.3 to 145ng/ml (mean 33.1; 95% CI: 11.3 to 54.9). Not all patients increased their GH level. The range of change from baseline was -17 to + 52ng/ml and group mean change from baseline was +10.8ng/ml (95% CI -1.7 to +23.3).

In the study of Jorgensen 2005 [19] (n = 11) PEG treatment alone more than doubled group mean GH levels. Subsequent combination of 15mg PEG/day with LASSA treatment (every two to four weeks) appeared to decrease GH levels and suppress some of the induced rise due to PEG (difference in group means not statistically significant). PEG may interfere with commercial kit-based immunoassays for GH Paisley et al 2007 [25], and this could impact on the quantitative interpretation of published results.

### Clinical effectiveness: adverse events and withdrawal from treatment

In the RCT [7] the 20mg/day PEG group experienced slightly higher rates of adverse events than the placebo group (Table 2). One patient withdrew from PEG because of persistent headache and another due to raised serum level of liver enzyme; one placebo patient also withdrew for persistent headache. The RCT extension [8], and studies of Schreiber [21], and Jehle [17] also reported adverse events (Table 2). The RCT extension [8] reported higher rates of adverse events than the RCT. Of 160 participants who received PEG thirty (19%) withdrew from treatment for various reasons (nine for adverse events, five for lack of efficacy, twelve "voluntarily", and two each were lost to follow up or "violated protocol"). Overall withdrawal in Schreiber [21] was unclear. In the 12 month study of Colao [16] four of 16 patients (25%) withdrew or were withdrawn, one each for: failure to inject PEG; raised liver enzyme level; inability to follow the protocol; poor compliance. The RCT extension [8] reported that liver enzyme activities in serum remained within normal range during PEG treatment. Schreiber [21] reported abnormally raised serum levels of liver enzymes in 21 of 229 (9%) patients; in 12 of these the levels were ≥ 3-fold above normal. Of the 12 with very elevated levels seven patients returned to normal during PEG treatment, levels returned to normal in four patients after withdrawal of PEG and in one patient level remained high but PEG was continued. Details of six of Schreiber's patients were reported by Biering 2006 [11]. In this report 6 of 142 (4%) withdrew permanently from PEG because of raised liver enzyme levels.

**Table 2:** Rate of adverse events reported in RCT and non-randomised studies

***Adverse event***	**Trainer 2000**[7] **Placebo** **n = 32**	**Trainer 2000**[7] **PEG 10mg/d** **n = 26**	**Trainer 2000**[7] **PEG 15mg/d** **n = 26**	**Trainer 2000**[7] **PEG 20mg/d** **n = 28**	**^¶ ^†Open label** **van der Lely (2001)**[8] **PEG** **n = 160**	**^¶¶ ^††Open label Scheiber (2007)**[21] **PEG** **n = 229**	**†††Open label Jehle (2005)**[17] **PEG** **n = 10**
*Infections*	5* (16%)	5* (19%)	4* (15%)	5* (18%)	52 (33%)		1 (10%)

*Headache*	4 (12%)	3 (12%)	2 (8%)	3 (11%)	41 (26%)	4 (1.7%)	3 (30%)

*Injection-site reaction*	0 (0%)	2 (8%)	1 (4%)	3 (11%)	18 (11%)	17 (7.4%)	--

*Pain*	2 (6%)	2 (8%)	1 (4%)	4 (14%)	36 (23%)	--	--

*Diarrhoea*	1 (3%)	1 (4%)	0 (0%)	4 (14%)	23 (14%)	--	--

*Nausea*	1 (3%)	0 (0%)	2 (8%)	4 (14%)	--	--	--

*Flatulence*	0 (0%)	0 (0%)	1 (4%)	3 (11%)	--	--	-v

*Influenza-like syndrome*	--	--	--	--	33 (21%)	--	--

*Accidental injury*	--	--	--	--	28 (18%)	--	--

*Hypercholesterolemia*	--	--	--	--	23 (14%)	--	--

*Back pain*	--	--	--	--	21 (13%)	--	--

*Asthenia*	--	--	--	--	21 (13%)	--	--

*Arthralgia*	--	--	--	--	19 (12%)	--	--

*Sinusitis*	--	--	--	--	16 (10%)	--	--

*Insomnia (transient)*	--	--	--	--	--	--	2 (20%)

*Fatigue*	--	--	--	--	--	--	3 (30%)

¶*Number of patients (%) with adverse events that occurred in at least 10% of patients*.

¶P *Number of patients (%) with adverse events occurring in > 1% and judged potentially causally related to PEG treatment*.

**† ***mean follow up 61 weeks*. **†† ***maximum follow up 102 weeks*. **††† **follow up 10 to 20 months. * upper respiratory tract infection only.

### Clinical effectiveness: miscellaneous outcome measures

A miscellany of further outcomes were reported mostly from retrospective laboratory analyses of serum samples for surrogate markers of disease-risk (diabetes, cardiovascular disease, maladjusted bone turnover) in subgroups of patients from other studies (mainly the RCT [7] and/or its extension [8]). Four studies [9,13,16,22] reported on statistical significance of before versus after PEG treatment changes in the levels of several risk markers for cardiovascular disease. The findings (Table 3) were not consistent between studies and no firm conclusions justifiable. One study reported statistically significant improvement in cardiac structure and function after 18 months of PEG treatment using Doppler echocardiography[20].

**Table 3:** Risk indicators for CVD: comparison for before v. after PEG There were small numbers of participants and changes for most markers did not reach statistical significance

	**Sesmilo 2002 **[9]**§** **n = 26**	**Colao 2006 **[16] **n = 16**	**Parkinson 2002 **[22]† **n = 20**	**Parkinson 2002 **[22]† **n = 20**	**Paisley 2006 **[13]* n = 20
**PARAMETER**	**Mean change from baseline *P***	**Paired t test** **Before** **v.** **after Tx *P***	**Paired t test Before** **v.** **after Tx *P***	**Cases:** **Before Tx** **v.** **after Tx *P***	**Cases before Tx** **v.** **Healthy controls *P***

Total chol (mM)	0.22 *NS*	*NS*	*Increased ****<0.01***	*Increased ****<0.01***	*0.16*

HDL chol (mM)	0.006 *NS*	*Increased ****0.0017***	*NS*	--	--

LDL chol (mM)	-0.13 *NS*	--	*Increased ****<0.01***	--	--

[Total/HDL] chol	0.21 *NS*	*Reduced ****0.0012***	--	--	--

TG (mM)	0.25 ***0.007***	*NS*	*NS*	*0.3*	*0.13*

Lipo *(a) *(mg/l)	-70 ***0.039***	--	*Reduced ****<0.01***	--	--

Apo B	--	--	*Increased ****<0.01***	--	--

Apo A1	--	--	*Increased ****<0.05***	--	--

Homocysteine (?M)	-0.16 *NS*	--	--	--	--

CRP	2 ***0.0002***	--	--	--	--

Interleucin 6	0.17 *NS*	--	--	--	--

Blood pressure	--	*NS*	--	--	--

Fibrinogen	--	*NS*	--	--	--

Heart rate	--	*NS*	--	--	--

MMP-2 (ng/ml)	--	--	--	*Reduced ****<0.001***	*Higher ****<0.001***

MMP-9	--	--	--	*0.76*	*0.87*

VEGF	--	--	--	*Reduced ****0.008***	*0.18*

§ results for the open label part of the study, patients included if they normalised IGF-1 with treatment. It is unclear if the number of patients analysed was 34 or 26. † Units for Total chol and total TG given in paper as mM but are actually mg/dl. * It is possible that some participants may have been used in both these studies.

Apo A1 = apoprotein A1 (on HDL & chylomicrons). Apo B = apoprotein B (on LDL). chol = cholesterol CRP = C-reactive protein. HDL = high density lipoprotein. LDL = low density lipoprotein. Lipo *(a) *= lipoprotein little a. MMP = matrix metalloproteinase. *NS *= not statistically significant. *P *= probability. PEG = pegvisomant. TG = triacyl glyceride. Tx = treatment. VEGF = vascular endothelial growth factor.

Note: There were small numbers of participants and changes for most markers did not reach statistical significance.

Two studies [10,15] presented data on serum markers of bone metabolism. The results (Table 4) support the proposition that PEG reduces bone turnover in acromegaly; how this translates to patient benefit requires further investigation. Several small non-randomised studies reported on laboratory measures relating to insulin and/or glucose metabolism; these included those of Barkan 2005 (n = 53) [24], Parkinson 2002 (n = 20) [22], Parkinson 2003b (n = 16) [23], Colao 2006 (n = 16) [16], Jehle 2005 (n = 10) [17], Jorgensen 2005 (n = 11) [19]. The general direction of findings was for a favourable change indicative of improved metabolic adjustment. The significance of these findings for patient well-being is difficult to judge.

**Table 4:** Indicators of bone formation and soft tissue turnover

**MARKER**	**Parkinson 2003 **[15]**Φ baseline**	**Parkinson 2003**[15]**Φ At IGF-1 normalisation**	**P v. baseline**	**Fairfield 2002**[10]**ΦΦ placebo 12 wks**	**Fairfield 2002**[10]**ΦΦPEG 12 wks**	**P v. placebo**
***Bone formation***						

osteocalcin	47^θ ^(14 - 109)	21 (10 - 73)	< 0.001	+0.01^θθ ^(0.39)	-2.2 (0.44)	0.009

terminal propeptide procollagen I	70^θ ^(12)	38 (8)	< 0.01	+18.1^θ ^(12.8)	-23.6 (9.6)	0.022

bone alkaline phosphatase	147^θθθ ^(29)	120 (23)	< 0.05			

***Bone resorption***						

cross linked telopeptide of collagen I	0.8^Ψ ^(0.2 - 2.4)	0.4 (.03 - 1.3)	<0.0001	+1^θθ ^(0.3)	-4.4 (1.4)	0.024

urinary ratio cross linked telopeptide/creatinine	92^θθθ^	56 (14)	< 0.01			

***Soft tissue formation***						

terminal propeptide of procollagen III	4.3^θ ^(0.3)	3.1 (0.3)	<0.01			

Φ data are mean (SEM) or median (range); ΦΦ data are mean (SEM); θ ug/L; θθ nmol/L; θθθ units unclear. Ψ pmol/L.

### Economic analysis

**Figure 4:**
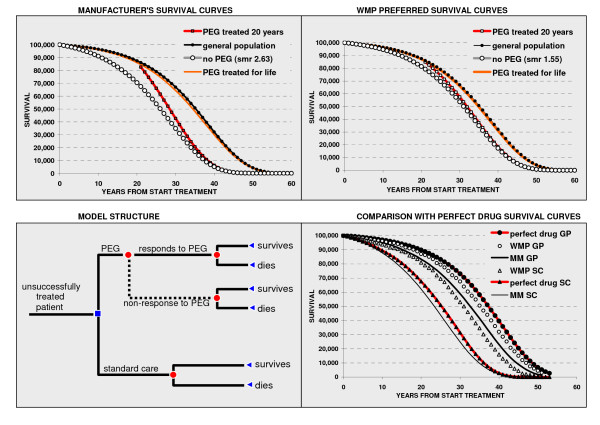
**Model structure and survival curves**. Manufacturer's (MM), Welsh Medicines Partnership (WMP) and perfect drug scenario (PD) input survival curves used in economic model. Data input shown as symbols, solid lines represent Gompertz distributions fit to data. In the MM and WMP models PEG treatment continues for 20 years and compliance is 92%. GP = general population (survival in PEG-compliant patients returns survival to that of the general population). SC = standard care (survival in standard care derived by applying a standardised mortality rate (SMR = 2.63 for MM, 1.5 for WMP, and 3.6 for PD) to the survival of the GP). The curves shown are modelled using mortality data from the Office of National Statistics for England (population about 50 million) or for Wales (population about 5 million) as appropriate.

One publication was included. This 2005 Technology Assessment [26] for the Welsh Medicines Partnership (WMP) described and critiqued a manufacturer's (Pfizer UK Ltd) submitted decision analytic model of PEG treatment versus standard care (SC) from the perspective of the UK NHS. The WMP re-ran the model using "preferred parameters". We were provided with a working version of the manufacturer's model (MM). Figure 4 shows the decision tree structure of the model.

### Description of Manufacturer's Model

The MM assumed SC to be treatment with long acting SSAs. It considered 100,000 English male patients starting treatment at an average age of 45 years and continuing PEG for at least 20 years. Benefits of PEG derived from improved survival and improved quality of life (QoL). The survival of SC patients (the comparator population) was obtained by applying a standardised mortality ratio for acromegaly (2.63, Bates 1993 [27]) to the National Statistics life table for English men aged 45 to 65 years. To calculate survival benefit it was assumed that 92% of PEG treated patients were responders and attained the survival probability of English males while 8% were non-responders and remained in PEG treatment with the survival probability of standard care (SC) patients. For those who stopped PEG treatment after 20 years survival probability at each subsequent year thereafter was the same as that for the SC survivors to that year. The resulting survival curves are shown in Figure 4. The survival benefit was calculated from the difference between survival curves. In the absence of pertinent data a utility gain in QoL was assumed to be equal to the disutility of patients experiencing a coronary event (0.83 - 0.75 = 0.08) and in the MM was experienced by all PEG treated patients (including the 8% non-responsive PEG-treated patients). The same gain was applied for each year of PEG treatment. With discounting at 3.5% for costs and benefits the MM delivered incremental cost-effectiveness ratios (ICERs) at 20 years of £105K/quality adjusted life year (QALY) and £194K/life year gained (LYG).

### Welsh Medicines Partnership's re-run of Manufacturer's Model

The WMP re-ran the MM using preferred parameters that included survival of PEG responders taken as that of a mix of Welsh men and women in proportion and with survival probability from National Statistics life tables; survival in SC was obtained by applying an SMR of 1.55 (Orme 1998 [28]). The impact of these changes on the difference in survival curves for PEG and SC treated individuals is shown in Figure 4. The ICER generated at 20 years was £748K/LYG.

### Current author's re-run of Manufacturer's Model

**Figure 5:**
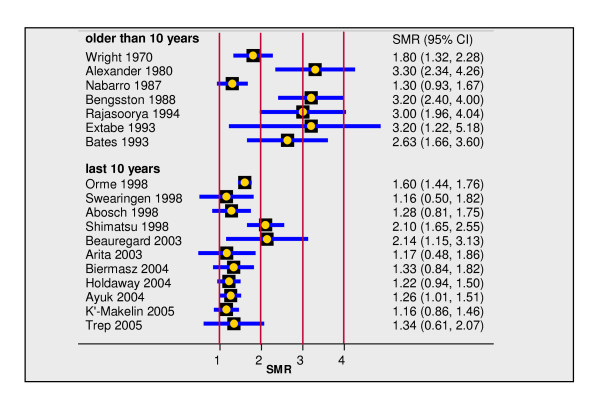
**SMR values reported in acromegaly studies**. Standardised mortality ratios reported in studies of patients with acromegaly, sorted according publication in last decade or earlier.

We estimated a feasible lower limit for the ICER of PEG treatment versus SC. To do this we adopted a "perfect drug" scenario in which model inputs were all in favour of PEG relative to SC but remained within reasonable bounds set by available information. We assumed complete compliance with PEG treatment and complete effectiveness so that survival and QoL were returned to those of the general population. This simplifies the model by eliminating the non-responder arm (dashed line in Figure 4) as it was unrealistic that non-responder patients would persist with PEG for 20 years. With regard to survival we adopted two further changes: first the general population was taken from National Statistics life tables for English men and women aged 45 onwards; second we applied a standardised mortality ratio of 3.6. We reasoned that survival of acromegaly patients eligible for PEG would likely be poorer than for the generality of acromegaly patients, such as those in Orme [28]. Thus, we assumed survival of patients eligible for PEG to be more like that observed in early studies before the development of long acting SSAs which normalise IGF-1 and GH in many patients. In addition many of the patients eligible for PEG will have had radiotherapy which is a known independent indicator of poorer survival [29,30]. We therefore examined standardised mortality ratios from studies of more than a decade ago (Figure 5). We increased the highest ratio from these studies by 10% giving a standardised mortality ratio for the perfect drug scenario of 3.6. The resulting survival curves are compared with the MM and WMP curves in Figure 4.

Several studies [31-36] have now reported poorer QoL for acromegaly patients relative to the general population; the direction of evidence from these points to a negative relationship between QoL and IGF-1 levels, but no studies have examined utility gain from PEG treatment. Rowles 2005 [31] reported utility values for QoL of acromegaly patients (72.5% had active disease, 27.5% were in remission). Median utility was 0.7 (range -0.07 to 0.92) compared to 0.81 for matched members of the general public. To allow for the fact that patients eligible for PEG would correspond to patients with active disease we approximated the utility of such patients as 0.66 (i.e. 0.81 - ([0.81-0.7] × [1/0.725])). The utility gain in the perfect drug scenario was therefore 0.15 (i.e. 0.81 - 0.66) compared to 0.08 in the MM.

The average dose of PEG/day for UK patients is 16.5 mg (Pfizer UK Ltd, personal communication). At £100/20 mg (BNF [37]) the average acquisition cost for PEG was taken as £30,133pa (assuming vial splitting as necessary). Additional cost for MRI scans and laboratory tests (IGF-1 and serum levels of liver enzymes) were £349pa (expert clinical opinion and NHS reference costs [38]). For the perfect drug scenario we assumed standard care patients were all treated with long acting SSAs; this assumption favours PEG since some patients are resistant to LASSAs and if given pharmacotherapy would receive much cheaper drugs (e.g. DOPAs). The MM used an annual acquisition cost for long acting SSAs of £13,289; other independent estimates range from £9,000 to £12,000 [39] and £7,000 to £14,000 [40]. We used £13,289pa. In addition, SC included costs for scans and laboratory tests (£349pa as above) and £1,771pa for treatments of co-morbidities (Didoni 2004 [41]). The total annual cost for PEG and for SC was £30,482 and £15,409 respectively.

At 20 and 40 years, with the inputs described above, the perfect drug scenario delivers ICERs of £81K/QALY and £212/LYG and £65K/QALY and £94K/LYG respectively. To reduce the ICER at 20 years to £30K/QALY would require a reduction in PEG cost by about one third.

## Discussion

With regard to the volume and quality of evidence on the effectiveness of PEG, difficulties in patient recruitment may partly explain the existence of only a single RCT, and the participation of overlapping populations of patients amongst multiple studies. Most non-randomised evidence came from "convenience" samples and these studies were susceptible to biases of patient selection and in some cases incomplete follow up. In general, study size was small and follow up was short with relatively little or unclear reporting about withdrawals from treatment. It is likely very few studies investigated the "licensed population". The RCT was conducted before licensing. In 2006 Colao [16] commented *"all these studies were not designed to investigate the response to pegvisomant in patients with proven resistance to long-term, high dose therapy with somatostatin analogues; these are the patients allowed to receive pegvisomant treatment in Europe according to the product label."*

Convincing evidence indicated that adequate dosage of PEG delivers significant reductions in IGF-1 levels and a substantial proportion of such patients are brought within normal range [7,8,16,18,21]. In contrast the levels of GH appear on average to be considerably increased by PEG treatment as shown in the twelve week RCT [7], the uncontrolled extension [8] and by two small non-randomised studies [16,19]. Individual patient data shows GH may not increase in all patients [16]. Tumour size is apparently unaffected by PEG treatment [8,16-19,21,24], at least in the short term, however continued vigilant monitoring of tumour size is mandatory during PEG treatment.

In the twelve week RCT [7] PEG improved patient scores for five signs and symptoms of disease (soft tissue swelling, arthralgia, headache, excessive perspiration and fatigue). The improvements reached statistical significance for tissue swelling, perspiration and fatigue. Several small non-randomised studies reported similar trends in improvement.

Increased mortality in acromegaly has been associated with cardiovascular problems. The effect of PEG upon risk indicators for cardiovascular disease was examined in several of the non-randomised studies included in this review but the results were not wholly consistent or easily interpreted. A single small 18 month non-randomised study [20] provided evidence that PEG induces favourable changes in cardiac structure and performance.

The limited available information indicates that PEG has a generally mild adverse event profile but occasionally raises liver enzyme levels necessitating temporary or very occasionally permanent withdrawal [16,21]. This means monitoring for possible liver damage is a necessity during long term administration of PEG. Other side effects include headache, injection-site reactions, flu-like syndrome and recently injection-site lipodystrophy sufficient to cause discontinuation has been reported [42]. Antibodies to PEG appear rarely to have been measured. Evidence is lacking about any relationship between anti-GH antibodies and decrease in efficacy or an increase in adverse events frequency.

The economic model with input parameters all favouring PEG relative to standard care (a perfect drug scenario) showed that over a 20 year time horizon the cost effectiveness of PEG is very unlikely to fall below £80,000/QALY or £212,000/LYG. In the absence of special criteria for the assessment of orphan drugs, this means that PEG is unlikely to represent good value for money when considered against the current standards applied to interventions in the UK Health service.

## Conclusion

PEG effectively reduces IGF-1 levels and improves signs and symptoms of acromegaly. Adverse events associated with treatment are of relatively minor severity and low prevalence, at least in the short term, and compliance is probably about 80%. When considered against the norms currently used to determine cost-effectiveness within the UK, PEG does not represent good value for money. For PEG treatment to be more acceptable to commissioners special criteria appropriate to orphan drugs would need to be adopted or the cost of PEG reduced.

## List of abbreviations

GH: growth hormone; IGF-1: insulin like growth factor-1; PEG: pegvisomant; QALY: quality adjusted life year; LYG: life year gained; GHRH: growth hormone releasing hormone; DOPA: dopamine agonist; SSA: somatostatin agonist; LASSA: long acting SSA; RCT: randomised controlled trial; ICER: incremental cost-effectiveness ratio; MRI: magnetic resonance imaging; CI: confidence interval; SC: standard care; MM: manufacturer's model; WMP: Welsh Medicines Partnership; BNF: British National Formulary; NHS: National Health Service; QoL: quality of life; PD: perfect drug; SMR: standardised mortality ratio.

## Competing interests

The authors declare that they have no competing interests.

## Authors' contributions

DJM conceived and supervised the study, helped draft and edited the manuscript. YA developed the protocol, extracted data on effectiveness and helped design the search strategies. MJC synthesised the effectiveness evidence, modified and implemented the economic model and helped draft the manuscript. SB designed and implemented the search strategies. All authors have read and approved the final manuscript.

## Pre-publication history

The pre-publication history for this paper can be accessed here:



## Supplementary Material

Additional file 1 **Search strategy details**. Provides details of search strategies used to identify relevant research Click here for file

Additional file 2 **Identification of effectiveness studies and list of excluded studies**. Provides a flow diagram of the selection of reviewed evidence and details of studies/articles that did not meet all of the selection criteria Click here for file

Additional file 3 **Main characteristics of the included publications**. Provides details of the characteristics of the reviewed studies Click here for file

Additional file 4 **Further details of included studies**. Provides further details of the reviewed studies Click here for file
