# Supplementary material for: Clinical effectiveness and cost-effectiveness of pegvisomant for the treatment of acromegaly: a systematic review and economic evaluation
Source: BMC Endocr Disord. 2009 Oct 8;9:20. doi: 10.1186/1472-6823-9-20 (PMC2768727; doi:10.1186/1472-6823-9-20)
Supplement: Additional file 1 — Search strategy details. Provides details of search strategies used to identify relevant research [file 1472-6823-9-20-S1.PDF]

DETAILS OF SEARCH STRATEGIES

EFFECTIVENESS. The following data bases were searched using the listed strategies.

Database: Cochrane Library (Wiley) 2007 Issue 1
Search strategy

#1 pegvisomant
#2 somavert
#3 growth next hormone next receptor next antagonist*
#4 b2063
#5 #1 or #2 or #3 or #4
#6 acromegaly
#7 exp acromegaly/
#8 #6 or #7
#9 #5 and #8

Database: MEDLINE(Ovid) 1950 to March Week 3 2007
Search strategy

1 pegvisomant.mp.
2 somavert.mp.
3 b2036.mp.
4 growth hormone receptor antagonist$.mp. or exp growth hormone receptor antagonist/
5 or/1-4
6 acromegaly.mp. or exp ACROMEGALY/
7 (systematic adj review$).tw.
8 (data adj synthesis).tw.
9 (published adj studies).ab.
10 (data adj extraction).ab.
11 meta-analysis/
12 meta-analysis.ti.
13 comment.pt.
14 letter.pt.
15 editorial.pt.
16 animal/
17 human/
18 16 not (16 and 17)
19 5 not (13 or 14 or 15 or 18)
20 or/7-12
21 19 and 20

Database: MEDLINE(Ovid) 1950 to March Week 3 2007
Search Strategy:

1 pegvisomant.mp.
2 somavert.mp.
3 growth hormone receptor antagonist$.mp.

4 b2036$
5 or/1-4
6 acromegaly.mp. or exp ACROMEGALY/
7 6 and 5

Database: MEDLINE (Ovid) In-Process & Other Non-Indexed Citations April 3 2007
Search Strategy

1 pegvisomant.mp.
2 somavert.mp.
3 growth hormone receptor antagonist$.mp.
4 b2036$
5 or/1-4
6 acromegaly.mp. or exp ACROMEGALY/
7 5 and 6

Database: EMBASE (Ovid) 1980 to 2007 Week 13
Search Strategy:

1 pegvisomant.mp. or exp PEGVISOMANT/
2 somavert.mp.
3 growth hormone receptor antagonist$.mp.
4 b2036$
5 or/1-4
6 acromegaly.mp. or exp ACROMEGALY/
7 5 and 6

Database: CINAHL - Cumulative Index to Nursing & Allied Health Literature (EBSCO) 1982 to April 4 2007
Search Strategy:

1 pegvisomant.mp.
2 somavert.mp.
3 growth hormone receptor antagonist$.mp.
4 b2036$
5 or/1-4

Additional sources searched for abstracts, proceedings and ongoing research:

ENDO 2005, 2006 (Endocrine Society's 87th and 88thAnnual Meeting 2005,2006) , ZETOC (British Library database including proceedings); NRR 2007 Issue 1, Clinical Trials.gov as at April 2007. Terms used taken from Cochrane Library search strategies.

Cost effectiveness searches :

Database: MEDLINE(Ovid) 1950 to March Week 3 2007
Search Strategy: Cost searches

1 pegvisomant.mp.

2 somavert.mp.
3 b2036$.mp.
4 growth hormone receptor antagonist$.mp. or exp growth hormone receptor antagonist/
5 or/1-4
6 acromegaly.mp. or exp ACROMEGALY/
7 5 and 6
8 economics/
9 exp "costs and cost analysis"/
10 cost of illness/
11 exp health care costs/
12 economic value of life/
13 exp economics medical/
14 exp economics hospital/
15 economics pharmaceutical/
16 exp "fees and charges"/
17 (econom$ or cost or costs or costly or costing or price or pricing or pharmacoeconomic$).tw.
18 (expenditure$ not energy).tw.
19 (value adj1 money).tw.
20 budget$.tw.
21 or/8-20
22 7 and 21

Database: MEDLINE(Ovid) 1950 to March Week 3 2007
Search Strategy: Economic modelling searches

1 pegvisomant.mp.
2 somavert.mp.
3 b2036$.mp.
4 growth hormone receptor antagonist$.mp. or exp growth hormone receptor antagonist/
5 or/1-4
6 acromegaly.mp. or exp ACROMEGALY/
7 5 and 6
8 decision support techniques/
9 markov.mp.
10 exp models economic/
11 decision analysis.mp.
12 cost benefit analysis/
13 or/8-12
14 7 and 10
15 7 and 13

Also searched : NHS EED Cochrane Library (Wiley) 2007 Issue 1, OHE HEED April 2007 Issue (see Cochrane Library search strategy for terms used)

Database: MEDLINE (Ovid) 1950 to March Week 3 2007
Search Strategy: Side effects /quality of life

1 pegvisomant.mp.
2 somavert.mp.
3 growth hormone receptor antagonist$.mp.
4 b2036$
5 or/1-4
6 acromegaly.mp. or exp ACROMEGALY/
7 5 and 6
8 side effect$.mp.
9 adverse effect$.mp.
10 adverse event$.mp.
11 or/8-10
12 7 and 11
13 quality of life/
14 life style/
15 health status/
16 health status indicators/
17 or/13-16
18 7 and 17
19 5 and 17
20 6 and 17
21 or/18-20

Database: EMBASE (Ovid) 1980 to 2007 Week 13
Search Strategy: Quality of life

1 acromegaly.mp. or exp ACROMEGALY/
2 quality of life.mp. or exp "Quality of Life"/
3 exp Lifestyle/
4 exp Health Status/
5 exp Health Survey/
6 or/2-5
7 1 and 6
