# Supplementary material for: Clinical effectiveness and cost-effectiveness of pegvisomant for the treatment of acromegaly: a systematic review and economic evaluation
Source: BMC Endocr Disord. 2009 Oct 8;9:20. doi: 10.1186/1472-6823-9-20 (PMC2768727; doi:10.1186/1472-6823-9-20)
Supplement: Additional file 2 — Identification of effectiveness studies and list of excluded studies. Provides a flow diagram of the selection of reviewed evidence and details of studies/articles that did not meet all of the selection criteria [file 1472-6823-9-20-S2.PDF]

IDENTIFICATION OF EFFECTIVENESS STUDIES AND LIST OF EXCLUDED STUDIES

1]

Flow diagram of identification of effectiveness studies.

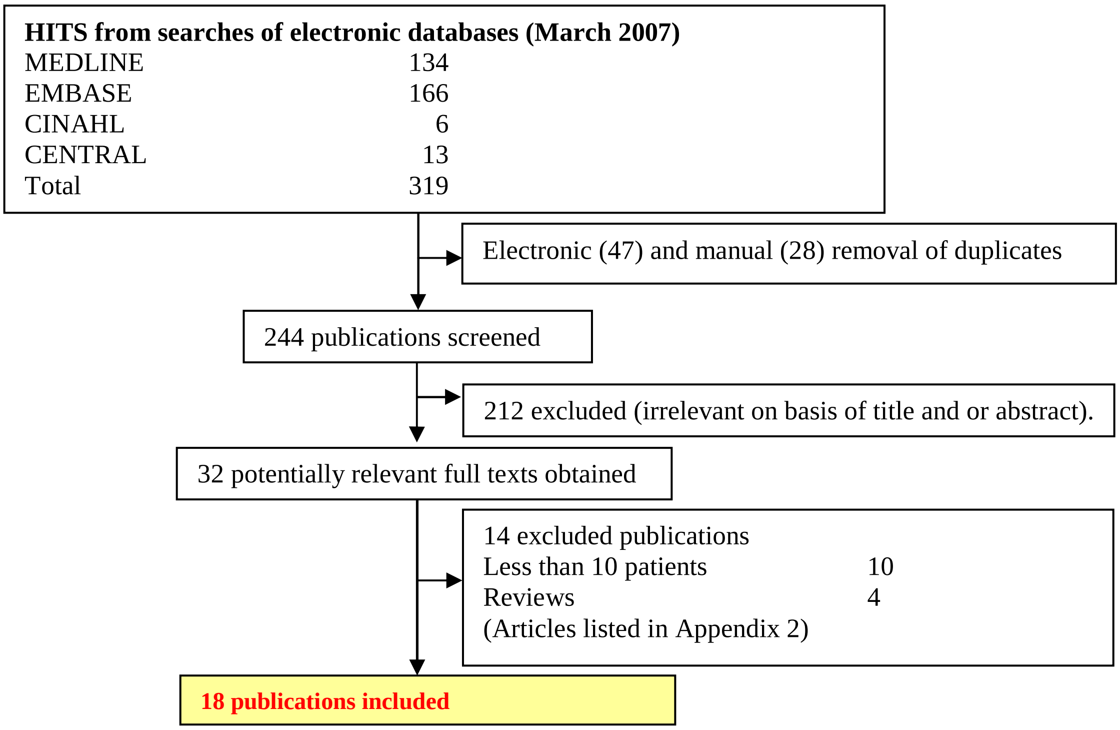

2] Table of studies excluded after examination of full texts with reasons for exclusion.

REFERENCE	REASON FOR EXCLUSION
Trainer PJ, Drake WM, Perry LA, Taylor NF, Besser GM, Monson JP. Modulation of cortisol metabolism by the growth hormone receptor antagonist pegvisomant in patients with acromegaly. The Journal of clinical endocrinology and metabolism 2001; 86(7):2989-2992	Less than 10 patients
Herman-Bonert VS, Zib K, Scarlett JA, Melmed S. Growth hormone receptor antagonist therapy in acromegalic patients resistant to somatostatin analogs. The Journal of clinical endocrinology and metabolism 2000; 85(8):2958-2961.	Less than 10 patients
Galland F, Kamenicky P, Affres H, Reznik Y, Pontvert D, Le BY et al. McCune-Albright syndrome and acromegaly: effects of hypothalamopituitary radiotherapy and/or pegvisomant in somatostatin analog-resistant patients. Journal of Clinical Endocrinology & Metabolism 2006; 91(12):4957-4961.	Less than 10 patients
Main KM, Sehested A, Feldt-Rasmussen U, Main KM, Sehested A, Feldt-Rasmussen U. Pegvisomant treatment in a 4-year-old girl with neurofibromatosis type 1. Hormone Research 2006; 65(1):1-5.	Less than 10 patients
Lansang C, Chitaia N, Simpson NE, Kennedy L, Lansang C, Chitaia N et al. Serum IGF-1 in treated acromegaly - how normal is "normal"? Pituitary 2005; 8(2):135-138.	Less than 10 patients

Muller AF, van der Lely AJ, Muller AF, van der Lely AJ. Pharmacological therapy for acromegaly: a critical review. Drugs 2004; 64(16):1817-1838.	Not primary study or systematic review
Colao A, Pivonello R, Cappabianca P, Auriemma RS, De Martino MC, Ciccarelli A et al. The use of a GH receptor antagonist in patients with acromegaly resistant to somatostatin analogs. Journal of Endocrinological Investigation 2003; 26(10 Suppl):53-56.	Not primary study or systematic review
Trainer PJ, Trainer PJ. Lessons from 6 years of GH receptor antagonist therapy for acromegaly. Journal of Endocrinological Investigation 2003; 26(10 Suppl):44-52.	Not primary study or systematic review
Drake WM, Rowles SV, Roberts ME, Fode FK, Besser GM, Monson JP et al. Insulin sensitivity and glucose tolerance improve in patients with acromegaly converted from depot octreotide to pegvisomant. European Journal of Endocrinology 2003; 149(6):521-527.	Less than 10 patients
Burt MG, Ho KK, Burt MG, Ho KKY. Comparison of efficacy and tolerability of somatostatin analogs and other therapies for acromegaly. . Endocrine 2003; 20(3):299-305.	Not primary study or systematic review
Rose DR, Clemmons DR, Rose DR, Clemmons DR. Growth hormone receptor antagonist improves insulin resistance in acromegaly. Growth Hormone & Igf Research 2002; 12(6):418-424.	Less than 10 patients
Drake WM, Parkinson C, Akker SA, Monson JP, Besser GM, Trainer PJ et al. Successful treatment of resistant acromegaly with a growth hormone receptor antagonist. European Journal of Endocrinology 2001; 145(4):451-456.	Less than 10 patients
van der Lely AJ, Muller A, Janssen JA, Davis RJ, Zib KA, Scarlett JA et al. Control of tumor size and disease activity during cotreatment with octreotide and the growth hormone receptor antagonist pegvisomant in an acromegalic patient. Journal of Clinical Endocrinology & Metabolism 2001; 86(2):478-481.	Less than 10 patients
Grottoli S, Gasco V, Mainolfi A, De GD, Ghigo E. Positive metabolic impact of treatment with pegvisomant in an acromegalic patient. Hormone Research 2007; 67 Suppl 1:174-176.	Less than 10 patients
