# Supplementary material for: Clinical effectiveness and cost-effectiveness of pegvisomant for the treatment of acromegaly: a systematic review and economic evaluation
Source: BMC Endocr Disord. 2009 Oct 8;9:20. doi: 10.1186/1472-6823-9-20 (PMC2768727; doi:10.1186/1472-6823-9-20)
Supplement: Additional file 3 — Main characteristics of the included publications. Provides details of the characteristics of the reviewed studies [file 1472-6823-9-20-S3.PDF]

Main characteristics of included publications

**STUDY**<br>*(Region)*<br>Study Design FUNDING	**N**<br>*Age [SD] yrs*<br>*male (%)*<br>[Φ]ACM duration<br>[range ; SD]	Prior treatments<br>(% of sample)	PEG dose mg/day[ΦΦ]<br>**Comparison**	Treatment duration<br>*(follow up)*	**MAIN OUTCOME MEASURES**
**Trainer 2000[7]**<br>*(Europe, US)*<br>RCT<br>INDUSTRY	**112***<br>*48 [14]*<br>*56%*<br>8 [8] yrs	Surgery 83<br>Rx 57<br>SSA 72<br>DopA 49<br>None 3	80 day 1, then<br>10 (n=26); 15 (n=26); 20 (n=28)<br>**PEG (n=80) vs Placebo (n=32)**	12 wks<br>*(12 wks)*	**IGF-1** (Free IGF-1, IGFBP-3).<br>**GH**<br>**Signs & symptoms; Finger-ring size**<br>**Tumor volume** (NMR)<br>**Adverse events**
**Van der Lely 2001[8]**<br>§ *(Europe, US)*<br>Uncontrolled before-after<br>INDUSTRY	**160**<br>*46 [14]*<br>*59%*<br>8 [8] yrs	Surgery 84<br>Rx 59<br>SSA 73<br>DopA 48<br>None NR	Start at 10, titrate to normal IGF-1 or maximum dose 40.‡<br>**Before vs after PEG**	Mean 425 days<br>*(maximum 18 months)*	**IGF-1**<br>**GH**<br>**Tumor volume** (NMR)<br>**Adverse events**<br>**Laboratory tests** liver enzymes
**Sesmilo 2002[9]** §**<br>*(Europe, US)*<br>Placebo controlled, subgroup ‡‡<br>Uncontrolled before-after.<br>INDUSTRY	**48**<br>*45 [2]*<br>*52%*<br>4.6 [8.2] yrs	NR / UC.	10 (n=12), 15 (n=10) 20 (n=12) or placebo 12 wks, titrate to normal IGF-1 or dose 35 (n=48).<br>**PEG vs Placebo**<br>**Before vs after PEG**<br>**X sectional v healthy**	18 months maximum<br>*(18 months maximum)*	**IGF-1**<br>**GH**<br>**CVD markers** (total chol; HDL-chol; LDL-chol; Total chol/HDL-chol; TG; Lipo *a*; homocysteine; glucose; insulin<br>**Inflammatory markers** C-reactive protein (n=34)
**Fairfield 2002[10§]**<br>*(Europe, US)*<br>Placebo controlled, subgroup ‡‡. INDUSTRY	**27**<br>*45.2 [2.3]*<br>*44%*<br>NR / UC.	NR / UC.	10 (n=7)<br>15 (n=6)<br>20 (n=7)<br>**PEG (n=20) vs Placebo (n=7)**	12 wks<br>(*12 wks)*	**Bone markers** (osteocalcin, carboxy terminal propeptide of procollagen type 1, cross-linked *N*-telopeptides of type 1 collagen).
**Parkinson 2002[22§]**<br>*(UK)*<br>Uncontrolled before-after.<br>INDUSTRY	**20**<br>*58.7 [28-79]*<br>*45%*<br>NR / UC.	Surgery 70<br>Rx 60<br>Medical only 15	12 wks at various doses, titrate to normal IGF-1<br>**Before vs after PEG**	NR / UC..<br>*(NR / UC.)*	**IGF-1**<br>**Lipoproteins** total chol; HDL-chol; LDL-chol; apo B; apo A1; TG; Lipo *a;*<br>**Laboratory tests (**glucose; insulin; insulin resistance).
**Parkinson 2003a[15] ****<br>§*(Europe)*<br>Uncontrolled before-after.<br>INDUSTRY	**16**<br>*52 θ*<br>*56%*<br>NR / UC.	Surgery 81<br>Rx 75<br>Medical only 19	12 wks (various doses), titrate to normal IGF-1, mean 20.<br>**Before vs after PEG;**<br>**(X sectional v healthy)**	Mean 7 months to normal IGF-1<br>*(mean 7 months)*	**IGF-1**<br>**Bone markers** (osteocalcin; CO_2- terminal propeptide pro-collagen type 1; *N* terminal propeptide procollagen types 1 & 3; X-linked *C*-terminal-telopeptides type 1 collagen; alkaline phosphatase; vitamin D; calcium; parathyroid hormone)
**Parkinson 2003b[23]**<br>§ *(UK one centre)*<br>Uncontrolled before-after<br>INDUSTRY	**16**<br>*52 θ*<br>*56%*<br>NR / UC.	Surgery 81<br>Rx 75<br>Medical only 19	12 wks various doses, titrate to normal IGF-1, mean dose 20.<br>**Before vs after PEG (at IGF-1 normalisation)**	Median 9 months to IGF-1 normalisation<br>*(median 9 months)*	**IGF-1**<br>**Serum leptin**<br>**Serum leptin receptor**<br>**Plasma insulin**<br>**Plasma glucose**
**Parkinson 2004[14§]**<br>*(Europe)*<br>Uncontrolled before-after.<br>INDUSTRY	**16**<br>*52θ [ra 27-58]*<br>*56%*<br>NR / UC.	Surgery & Rx NR / UC<br>SSA 31<br>DopA 50	12 wks at various doses, titrate to normal IGF-1; median dose 15.<br>**Before vs after PEG; (X sectional v healthy**)	Mean 7 months to IGF-1 normal<br>*(mean 7 months)*	**Serum IGF-1**<br>**Serum IGFBP-1,-2,-3**
**Barkan 2005[24§]**<br>*(Europe, US)*<br>Uncontrolled before-after<br>UNCLEAR	**53**<br>*49 [23-81]*<br>*51%*<br>NR / UC.	Surgery 83<br>SSA 100<br>DopA 8<br>Rx 71<br>PEG 91	10 start 4wks after last SSA. Titrated at wk 12, 20, 28 according to IGF-1 level.<br>**Before vs after PEG**	32 wks<br>*(32 wks)*	**IGF-1**<br>**Plasma glucose**<br>**Tumour volume** NMR<br>**Safety** Gall bladder ultrasound
**Jehle 2005[17]**<br>*(US)*<br>Uncontrolled before after<br>INDUSTRY, NIH US	**10**<br>*50 [39-67]*<br>*70%*<br>8.6 [1-24]	Surgery 100<br>Rx 30<br>Medical 80<br>PEG 20	40 day 1, titrate to normal IGF-1& frequency adjusted to least for normal IGF-1.<br>**Before vs after PEG**	12 to 20 months<br>*(12-20 months)*	**IGF-1**<br>**Signs & symptoms; Finger-ring size Tumor volume** (NMR)<br>**Adverse events**<br>**Laboratory tests** BMI, blood pressure, glucose, insulin *etc*
**Jorgensen 2005[19]**<br>*(Denmark)*<br>Uncontrolled before after<br>NR / UC.	**11**<br>*46 [23-71]*<br>*64%*<br>NR / UC.	Surgery 82<br>Rx 45 SSA. 91	10 for 6 wks then 15 for 6 wks then 15 + SSA for 12 wks<br>**Before vs after PEG**	24 wks<br>*(24 wks)*	**IGF-1 (**total, free & bio-active)<br>**GH & PEG**<br>**Tumor volume** (NMR)<br>**Laboratory test** (glucose, insulin)<br>**Safety** (liver enzymes)
**Feenstra 2005[18]**<br>*(Holland)*<br>Uncontrolled before after<br>NR / UC.	**26§§**<br>*51 [13]*<br>*58%*<br>NR / UC.	Surgery 31<br>Rx 16<br>SSA 100	Long acting SSA (monthly) + PEG once / wk titrated from 25 mg until normalisation of IGF-1.<br>**Before vs after PEG**	42 wks<br>*(42 wks)*	**IGF-1**<br>**PEG (**weekly dose for IGF-1 normalisation)<br>**Safety** (liver enzymes)<br>**Tumor volume** (NMR)

**STUDY**<br>*(Region)*<br>Study Design FUNDING	**N**<br>*Age [SD] yrs*<br>*male (%)*<br>**Φ**ACM duration<br>[range ; SD]	Prior treatments<br>(% of sample)	PEG dose mg/day[ΦΦ]<br>**Comparison**	Treatment duration<br>*(follow up)*	**MAIN OUTCOME MEASURES**
**Paisley 2006[13]**<br>*(UK)*<br>Uncontrolled before after.<br>INDUSTRY, EU	**20**<br>*56 [14]*<br>*55%*<br>NR / UC	Surgery 80<br>Rx 80<br>SSA UC.	80 day 1, then 10, then increased every 8 wks until IGF-1 normalised. Mean 18.<br>**Before vs after PEG & (X sectional v healthy)**	Mean 6.5 months (ra 1-16)<br>*(NR / UC.)*	**IGF-1**<br>**CVD markers (**Matrix metallovascular proteinase, endothelial growth factor, Total chol:, TG, glucose).
**Biering 2006[11]**<br>*(Germany)*<br>Retrospective case series<br>NR / UC.	**142**<br>*NR / UC.*<br>*NR / UC.*<br>NR / UC.	NR / UC.	Not reported<br>**Observed vs normal**	Mean 28 wks [SD 20]<br>*(NR / UC.)*	**Safety (**liver enzymes)
**Colao 2006[16]**<br>*(Italy)*<br>Uncontrolled before after<br>INDUSTRY	**16**<br>*46θ (28-61)*<br>*47%*<br>NR / UC	Surgery 87<br>Rx 12<br>SSA 100<br>DopA UC	40 on day 1, then 10 rising by 5 every 6 wks until IGF-1 normalised or 40 reached.<br>**Before vs after PEG**	12 months<br>*(12 months)*	**GH**<br>**IGF-1**<br>**Signs & symptoms**<br>**Tumor volume** (NMR)<br>**Finger-ring size**<br>**CVD markers** (blood pressure; total chol; Total chol / HDL-chol; TG; fibrinogen; glycosylated hemoglobin; glucose; insulin)<br>**Safety** (liver enzymes)
**Pivonello 2007[20]**<br>*(Italy)*<br>Uncontrolled before after<br>INDUSTRY	**17§§§**<br>*48θ (27-61)*<br>*47%*<br>NR but ≥ 6 months	Surgery 82<br>Rx 12<br>SSA 82<br>DopA UC	40 on day 1, then 10 rising by 5 every 6 wks until IGF-1 normalised or 40 reached.<br>**Before vs after PEG**	6–18 months<br>*(6–18 months)*	**GH**<br>**IGF-1**<br>**Echocardiography** (LV mass, LV mass index; ejection fraction; LV posterior wall thickness; interventricular septum thickness);<br>**CVD markers** (blood pressure; total chol; Total chol / HDL-chol; TG; fibrinogen;glucose; insulin; HOMA; BMI).
**Schreiber 2007[21]**<br>*(Germany)*<br>Uncontrolled before after<br>INDUSTRY	**177§§§§**<br>*θθ40.5 [12.7]*<br>*47%*<br>Mean 9.1 yrs	Surgery 90<br>Rx 43<br>Medical 94	Mean 16.5 [SD 7.7] (94% pnts 10 to 30).<br>**Before vs after PEG**	Maximum 2 yrs<br>*(Maximum 2 yrs)*	**IGF-1**<br>**Signs & symptoms**<br>**Tumour volume** (NMR)
**Parkinson 2007[12]**<br>*(Europe, US)*<br>Retrospective<br>Uncontrolled before-after.<br>NR / UC.	**118§§§§§**<br>*44θ (27-61) 58%*<br>Mean 9.1 yrs	Rx 58	80 on day 1, then 10 titrating every 8 wks by 5 until iGF-1 normalised<br>**Before vs after PEG**	Mean 12 months [SD 7]<br>*(12 months SD 7)*	**IGF-1 (**Influence of baseline IGF-1 & other factors).

Φ time since diagnosis. **ΦΦ** unless stated otherwise. **θ** median. **θθ** age at diagnosis. § Study population wholly or mostly of participants from the RCT of Trainer 2000[7]. §§ only 19 of 26 participants analysed at 42 weeks. §§§ 14 of 17 patients were also participants in Colao 2006[16]. §§§§ Eligible population 229, 52 not evaluable. §§§§§ Eligible population 147, 29 not evaluable. * Patients excluded from the study if treated with a long-acting SSA within 12 weeks before enrolment. ‡ Most patients were those entered into the RCT of Trainer; the description of initial dosing regimen inconsistent. ‡‡ Subgroup of participants from Trainer, subgroup defined by study centre not by patient characteristics. ** In part of this study US patients from Trainer were analysed, stratification by centre may have allowed proper randomisation but this is not clear. In part of this study (cross sectional comparison) patients were compared to matched healthy controls. Unclear if prospective or retrospective (ie. post hoc) analysis of available serum samples. ¥ Describes patients with raised transaminase levels that were participants in study by Schreiber[21]. ALS: acid-labile subunit of IGFBP-3. BMI: body mass index. Chol: cholesterol. CVD: cardiovascular disease. DopA: dopamine analogue. HDL: high density lipoprotein. HOMA: homeostatic model adjustment index. IGFBP: IGF binding protein. LDL: low density lipoprotein. Lipo a: lipoprotein little a. LV: left ventricle / left ventricular. NMR: nuclear magnetic resonance scan. PEG: pegvisomant. pnts: patients. Rx: radiotherapy. SSA: somatostatin analogue. TG: triglyceride.
