# Supplementary material for: Clinical effectiveness and cost-effectiveness of pegvisomant for the treatment of acromegaly: a systematic review and economic evaluation
Source: BMC Endocr Disord. 2009 Oct 8;9:20. doi: 10.1186/1472-6823-9-20 (PMC2768727; doi:10.1186/1472-6823-9-20)
Supplement: Additional file 4 — Further details of included studies. Provides further details of the reviewed studies [file 1472-6823-9-20-S4.PDF]

Further details of studies

Study / Reference Region / Country	Design of Study / Country	Population with acromegaly					Intervention *subcutaneously daily unless otherwise stated*	Comp.	F/up	Main Outcomes
		**Inclusion Criteria**	**Exclusion Criteria**	**N**	**Previous Treatments (% by group)**	**Age / sex / other reported**				
Trainer 2000[7]<br><br>(Germany, Netherlands, Sweden, UK, and US)	Multicentre, Double-blind, placebo-controlled RCT of three different daily doses of pegvisomant (10, 15, or 20 mg) and placebo	Diagnosis of acromegaly on basis of signs and symptoms, pituitary adenoma on computed tomography or magnetic resonance imaging (MRI), and high IGF-1<br><br>Serum IGF-1 concentration at the second screening visit (off any previous medications for acromegaly) at least 1.3 times the upper limit of the age-adjusted normal range	Treatment with a long-acting somatostatin analog within 12 weeks before enrolment	112	Surgery:<br>Placebo / 10 mg / 15 mg / 20 mg<br>26(81%) / 22(85%) / 22(85%) / 23(82%)<br><br>Radiotherapy<br>placebo / 10 mg / 15 mg / 20 mg<br>17(53%) / 11(42%) / 14(54%) / 15(54%)<br><br>SS analogue<br>placebo / 10 mg / 15 mg / 20 mg<br>17 (53%) / 15(58%) / 9 (35%) / 14(50%)	*Mean Age:*<br>placebo / 10 mg / 15 mg / 20 mg<br>50 / 47 / 46 / 48<br><br>*Sex M/F*<br>placebo / 10 mg / 15 mg / 20 mg<br>19/13 / 15/11 / 14/12 / 15/18<br><br>*Duration of Acromegaly:*<br>Placebo / 10 mg / 15 mg / 20 mg<br>8±8y / 8±7 / 8±7 / 8±7	*N=26, 26, 28*<br><br>*Drug:*<br>pegvisomant<br><br>*Dose*<br>*10, 15, 20* mg daily<br><br>*Regime*, day 1 an 80 mg loading dose	*placebo*<br>*N = 32*	12 weeks	- Percentage change in serum IGF-1 concentration from base line.<br><br>- Free IGF-1, growth hormone (GH), IGF binding protein-3 (IGFBP-3), acid-labile subunit of IGFBP-3, ring size of the fourth (or fifth, if fourth finger too large) digit of right hand, scores for signs and symptoms (0 = No symptoms; 8 = Severe, symptoms)<br><br>- Anti-GH antibodies,<br>- hematology, serum chemistry, urinalysis,<br><br>- adverse events,<br>- tumor volume determined on MRI of pituitary, electrocardiogram
Van der Lely 2001[8]<br><br>(Germany, Netherlands, Sweden, UK, US)<br><br>This study is a subset of Trainer 2000	This study is an extension to the Trainer 2000 but it is now an open-label, uncontrolled, observational, dose-titration study following placebo-controlled clinical trials	Serum IGF-1 concentration at least 1.3 times the upper limit of the age-adjusted normal range at the second screening visit (at least 2 weeks after discontinuation of somatostastin analogs and at least 5 weeks after discontinuation of dopamine agonists)	None stated	167	treatment / 6 mo / 12 mo / 18 mo<br>surgery / 111(85) / 82(91) / 35(90)<br>X ray / 78(60) / 57(63) / 26(67)<br>SS / 97(74) / 74(82) / 33(85)<br>DA / 67(51) / 48(53) / 19(48)	*Mean Age*<br>*6 mo n=131* / *12 mo n=90* / *18 mo n=39*<br>*46±14* / *44±13* / *42±13*<br><br>*% M Sex*<br>*6 mo n=131* / *12 mo n=90* / *18 mo n=39*<br>*75(57)* / *47(52)* / *18(46)*<br><br>*Duration of Acromegaly*:<br>*6 mo n=131* / *12 mo n=90* / *18 mo n=39*<br>*8(8)* / *8(7)* / *8(8)*	*N=167 but only 152 received daily dosing*<br><br>*Drug pegvisomant*<br><br>*Dose*<br>Pegvisomant dose, mean ± SE (mg/d)<br>*6 mo n=131* 14.7 ± 0.4<br>*12 mo n=90* 18.0 ± 0.7<br>*18 mo n=39* 19.6 ± 1.4	*NA*	18-month	- Mean serum IGF-1 and GH concentrations at baseline<br><br>-Mean pituitary volume<br><br>-Fasting serum insulin concentrations<br>- Adverse events

Study / Reference Region / Country	Design of Study / Country	Population with acromegaly					Intervention *subcutaneously daily unless otherwise stated*	Comp.	F/up	Main Outcomes
		**Inclusion Criteria**	**Exclusion Criteria**	**N**	**Previous Treatments (% by group)**	**Age / sex / other reported**				
Fairfield 2002[10] Multicentre trial<br><br>This study is a subset of Trainer 2000	RCT placebo controlled but serum was available from Trainer 2000	Aged 18 years or more, clinical symptoms and signs of acromegaly, radiographic evidence of pituitary adenoma and IGF greater than 1.3 times the upper limit of age and sex-specific normal range.	Exclusion if did not meet:<br>2 weeks discontinuation of short SS, 5 weeks discontinuation dopamine agonist, of 12 weeks after discontinuation long acting SS	27	Not reported	*Mean Age*<br>*45.2±2.7 pegvisomant vs 45.2±5.1*<br><br>*F/M Sex: 10/10 in pegvisomant vs 2/5 placebo*<br><br>*Duration of Acromegaly*: not stated	*N=20*<br><br>*Drug pegvisomant*<br><br>*Dose 10, 15, 20 mg / day*	*vs placebo N=7*	12 weeks	Serum markers of bone turnover comparing patients taking 10, 15, 20 mg pegvisomant to placebo
Sesmilo 2002[9] Multicentre trial<br><br>This study is a subset of Trainer 2000	Cross sectional<br><br>Placebo controlled non random<br><br>Then longitudinal study after 12 weeks	Patients included following standard clinical and biochemical criteria and confirmed by imaging technique. IGF-I is 30% or greater above matched above the adjusted upper limit of the normal range.		47	Not stated	*Mean Age*<br>*45.±12 pegvisomant vs 45.±10*<br><br>*F/M Sex: 23/25 ve 26/18 control*	*Daily pegvisomant for 12 weeks for the RCT*<br>10 mg */ day* n=14, 15 mg */ day* n=10, 20 mg n =12.<br><br>However Subsequently all patients receive at least 10 mg */ day* pegvisomant for 18 weeks.	*47 matched for age and body mass index in healthy controls*<br><br>before vs after	12 weeks for RCT<br><br>18 months for the open lable	Cardio vascular risk factors before and after normalization of IGF-I<br>GH, IGF-I<br>Total Cholesterol<br>HLD, LDL<br>Total chol/HDL chol<br>Triglyceride, CRP<br>IL-6<br>Lipoprotien<br>Homocysteine<br><br>Glucose<br>Insuline<br>IRHOMA
Barkan 2005[24]	A multicentre open label trial<br>Before and after gesign	Patients with acromegaly previously treated with octreotide long-acting release.	Pituitary adenoma within 3mm of the optic chiasm, severe symptomatolog y that require surgery known or suspected alcohol abuse	53	Surgery:<br>83%<br><br>Radiotherapy<br>60%<br><br>SSA 100%, DopA 8%, PEG 91%	*Mean age* [yr(range)] 49(23-81)<br>Men/women[no.(%)] 27/26(51/49)	Pegvisomant 10mg /day	Measures at baseline week 0 taking octerotide	32 weeks	Glucose homeostasis<br>Effects on IGF-I and GH<br>Tumour volume<br>Safety

Study / Reference Region / Country	Design of Study / Country	Population with acromegaly					Intervention *subcutaneously daily unless otherwise stated*	Comp.	F/up	Main Outcomes
		Inclusion Criteria	Exclusion Criteria	N	Previous Treatments (% by group)	Age / sex / other reported				
Jorgensen 2005[19]	uncontrolled non randomised trial taking five different regimes following fixed treatment algorithm	Patients with acromegaly not responding adequately to conventional therapy	Not stated	11	Surgery: 82%<br>Radiotherapy 45%<br>SSA 91%	*Mean age* [yr(range)] 46 (23-71)<br>4 women , 7males	10 mg /day pegvisomant for 6 weeks.<br>Then 15 mg treatment with pegvisomant for 6 weeks.<br>Then 15 mg /d plus SMS for 12 weeks	SMS therapy alone.<br>Off SMS therapy for 2 months	Sequential duration of 5 different regimes 2 months no treatment with SMS, 6 weeks with 10 mg /d PEG, 6 weeks treatment with 15 mg/d ;12 wks SMS + PEG 15 mg/d.	Fasting glucose and glucose tolerance test<br>IGF-I<br>GH
Feenstra 2005[18]	Prospective Open label<br>Single centre.<br>Before and after combined therapy	Patients with active acromegaly who are not controlled with long acting SMS analogue	Not stated	26	Surgery: 15%<br>Radiotherapy & surgery 31%%<br>Neither radiotherapy or surgery 54%	*Mean (SD, range) 51 (12.6, 31-79)*<br>*Male 15(58%)*	Long acting SSA (monthly) + PEG once / wk titrated from 25 mg per week until normalisation of IGF-1 or a weekly dose reached 80 mg.	Somatostatin before combined therapy	42 weeks	IGF-I<br>Liver enzymes
Parkinson 2003a[15]<br>This study is a subset of Trainer 2000	Controlled non RCT	15 patients with an establishes diagnosis acromegaly were taken from an RCT (Trainer 2000) And one from van der Lely 2001)	Not stated	16	Surgery: 81%<br>Radiotherapy 75%	Median age 52 yr range [28-78y]<br>Male 9/16	10 mg */ day* pegvisomant with dose increments of 5 mg */ day* every 8 weeks until serum IGF-1 was in the age related reference range<br>Median dose 20 mg/d range 10-40 mg */ day*	32 age and sex matched ambulatory individuals	Mean 7 months<br>Range [3-11]	IGF-I<br>Markers of bone turnover:<br>PIIINP<br>OC<br>CTx<br>PINP<br>BAP<br>Tx/Cr ratio

Study / Reference Region / Country	Design of Study / Country	Population with acromegaly					Intervention *subcutaneously daily unless otherwise stated*	Comp.	F/up	Main Outcomes
		Inclusion Criteria	Exclusion Criteria	N	Previous Treatments (% by group)	Age / sex / other reported				
Parkinson 2003b[23]<br><br>This study is a subset of Trainer 2000	Before and after design	15 patients with an establishes diagnosis acromegaly were taken from an RCT (Trainer 2000) & one from van der Lely 2001)	Not stated	16	Surgery:<br>81%<br><br>Radiotherapy<br>75%	Median age 52 yr range [28-78y]<br>Male 9/16	10 mg */ day* pegvisomant with dose increments of 5 mg*/ day* every 8 weeks until serum IGF-1 was in the age related reference range	*Measures at baseline*	Mean 7 months<br>Range [3-11]	Serum leptin<br><br>Fasting plasma insulin<br><br>Fasting plasma glucose
Parkinson 2002[22]<br><br>(UK)<br><br>Most a subset of Trainer 2000	Two centres,<br><br>Before and after design	Diagnosis of acromegaly<br><br>Serum IGF-1 at least 1.3 x the upper limit of the age-adjusted normal range<br><br>SSA and DopA washout 2 and 5 weeks	Treatment with lipid lowering drugs	20	Surgery:<br>70%<br><br>Radiotherapy<br>60%<br><br>Medical only<br>15%	*Mean Age: yrs*<br>mean 58.7 range 28 to 79<br><br>*Sex M/F*<br>45% / 55%<br><br>*Duration of Acromegaly:*<br>Unclear	from 10 mg / day to normalisation of IGF-1 (dose change every 8 weeks as necessary)	baseline vs after IGF-1 normal	mean duration 10 months	base line. vs at normalisation of IGF-1<br><br>- serum IGF-1<br><br>-total chol; HDL-chol; LDL-chol; apo B; apo A1; TG;<br>Lipo *a;*<br><br>glucose; insulin; insulin resistance.
Parkinson 2004[14]<br><br>(UK)<br><br>15 of 16 a subset of Trainer 2000	Single centre,<br><br>Uncontrolled before and after design	Diagnosis of acromegaly<br><br>Serum IGF-1 at least 1.3 x the upper limit of the age-adjusted normal range<br><br>SSA and DopA washout 2 and 5 weeks		16	Surgery:<br>NR<br><br>Radiotherapy<br>NR<br><br>Just prior to PEG<br>SSA 31% DopA 50%	*Median Age: yrs*<br>median 52 range 27 to 58<br><br>*Sex M/F*<br>56% / 44%<br><br>*Duration of Acromegaly:*<br>Unclear	from 10 mg / day to normalisation of IGF-1 (dose change every 8 weeks as necessary)<br><br>mean dose 15 mg / day<br>range 10 to 40	baseline vs after IGF-1 normal	mean duration 7 months range 3 -11	base line. vs at normalisation of IGF-1<br><br>IGF-1<br><br>IGF binding proteins 1 , 2 & 3<br><br>tertiary complex-associated IGFBP-3
Jehle 2005[17]<br><br>(US)	Single centre,<br><br>Uncontrolled before and after design	Diagnosis of acromegaly and serum IGF-1 not normalised by SSA therapy.<br><br>Medications for acromegaly withdrawn at least 4 wks prior to PEG		10	Surgery:<br>100%<br><br>Radiotherapy<br>30%<br><br>Medical<br>SSA / Dop A 80% PEG 20%	*Age: yrs*<br>mean 50 range 39 to 67<br><br>*Sex M/F*<br>70% / 30%<br><br>*Duration of Acromegaly:*<br>Mean 8.6 years range 1-24	40 day 1, then 10 mg / day & then titrated from 10 until IGF-1 normal, then frequency adjusted to least needed for stable normal IGF-1. Before vs After. Mean dose 15 mg / day<br>range 10 to 40	dose repeats required for IGF-1 normal vs baseline	duration range 12 - 20	base line.<br><br>and dose frequency for normalisation of IGF-1

Study / Reference Region / Country	Design of Study / Country	Population with acromegaly					Intervention *subcutaneously daily unless otherwise stated*	Comp.	F/up	Main Outcomes
		**Inclusion Criteria**	**Exclusion Criteria**	**N**	**Previous Treatments (% by group)**	**Age / sex / other reported**				
Paisley 2006[13] (UK)<br><br>Cases a subset from Trainer 2000	Probably single centre,<br><br>before and after design<br><br>case (n=20) : control (n=25) study	Diagnosis of acromegaly<br><br>Serum IGF-1 at least 1.3 x the upper limit age-adjusted normal range<br><br>SSA and DopA washout 2 and 5 weeks	Treatment with a long-acting somatostastin analog	20	Surgery:<br>80%<br><br>Radiotherapy<br>80%<br><br>SSA<br>unclear	*Age: yrs*<br>*Acromegaly*: mean 56.1, SD 13.8; *control subjects*: mean 56.6, SD 13.8<br><br>*Sex M/F*<br>*Acromegaly* 55% / 45%; *control subjects* 52% / 48%<br><br>*Duration of Acromegaly:*<br>Unclear	*Regime* daily, subcutaneously. Day 1: 80 mg loading dose, then from 10 mg / day titrated every 8 wks by 5 mg / day as necessary to provide IGF-1 normality.<br><br>Dose range 10 to 60 mg / day	baseline vs after IGF-1 normal<br><br>cases vs controls	mean duration not reported<br><br>*"up to more than one year"*	cases vs controls for base line. vs at normalisation of IGF-1<br><br>Serum IGF-1<br>CVD markers**:** Matrix metallo- proteinase, endothelial growth factor, Total chol:, TG, glucose.
Colao 2006[16]<br><br>(Italy)	Probably single centre,<br><br>Uncontrolled before and after design	Acromegaly not responding to SSA. Serum IGF-1 at least 1.3 x the upper limit of the age-adjusted normal range.<br><br>SSA washout 4 months	Treatment with DopA within 5 weeks of study start, hepatitis, drug abuse, pregnant or nursing women	16	Surgery:<br>87%<br><br>Radiotherapy<br>12%<br><br>SSA 100%<br>DopA unclear	*Age: yrs*<br>median 46, range 28-61<br><br>*Sex M/F*<br>47% / 53%<br><br>*Duration of Acromegaly:*<br>Unclear / not reported	Day 1 40 mg loading dose, then 10 mg / day titrated every 6 wks by 5 mg / day as necessary to normalise IGF-1 (max: 40 mg / day)<br><br>Dose / day<br>Mean 23.7 [SD 9.7]<br>Median 25<br>Range 10 to 40	baseline vs after 12 [illegible]nths PEG	12 months	Base line. vs after 12 months PEG at dose required to normalise of IGF-1<br><br>Serum IGF-1 & GH<br>Tumour size (MRI)<br>Blood PEG levels<br>Side effects / liver enzymes<br>Signs and symptoms / ring size<br>CVD markers (blood pressure; total chol; total chol /HDL-chol; TG; fibrinogen; glycosylated Hb; glucose; insulin ; HOMA
Biering 2006[11]<br><br>(Germany)	multicentre<br><br>retrospective case series	Acromegaly, receiving PEG march 2003 to end 2004. "Most" only treated with PEG if failed to normalise IGF-1 with SSA	Not reported	142	Not reported	Not reported	Dose not reported<br><br>Mean dose duration 28.3 mg */ day* [SD 19.9] weeks.	baseline vs treatment	Max 21 months	Analysis of 12 patients that developed raised (more than 3 x normal) serum levels of liver transaminase enzymes.

Study / Reference Region / Country	Design of Study / Country	Population with acromegaly					Intervention *subcutaneously daily unless otherwise stated*	Comp.	F/up	Main Outcomes
		Inclusion Criteria	Exclusion Criteria	N	Previous Treatments (% by group)	Age / sex / other reported				
Pivonello 2007[20]<br><br>(Italy)<br><br>Mostly same patients as Calao 2006	Probably single centre,<br><br>Uncontrolled before and after design	Acromegaly not responding to SSA. Serum IGF-1 at least 1.3 x upper limit age-adjusted normal range.<br><br>SSA washout 4 months<br><br>Stable adenoma size for at least 12 months	Treatment with DopA within 5 weeks of study start. Hepatitis, drug abuse, pregnant or nursing women	17	Surgery:<br>82%<br><br>Radiotherapy<br>12%<br><br>SSA 100%<br>DopA unclear	*Age: yrs*<br>median 48, range 27-61<br><br>*Sex M/F*<br>47% / 53%<br><br>*Duration of Acromegaly:*<br>Unclear but> 6 months	Day 1 40 mg loading dose, then 10 mg / day titrated every 6 wks by 5 mg / day as necessary to normalise IGF-1 (max: 40 mg / day)<br><br>Dose / day<br>Mean 23.8 [SD 10.1]<br>Median 25<br>Range 10 to 40	baseline vs after 6 and 18 months PEG	Maximum 18 months	Serum IGF-1<br>Serum GH<br>PEG dose for normalisation of IGF-1
Schreiber 2007[21]<br><br>(Germany)	Multi centre,<br><br>"*Observational study*", uncontrolled before and after design<br><br>52 of 229 not evaluable	Not reported other than receiving PEG in Germany.	Not reported	229	Surgery:<br>90%<br><br>Radiotherapy<br>43%<br><br>204 (89%) received but stopped SSA (octreotide); 139 uncontrolled IGF-1,<br>23 complications of treatment12 for both reasons.	*Age AT DIAGNOSIS: yrs*<br>mean 40.5, SD 12.7<br><br>*Sex M/F*<br>47% / 53%<br><br>*Duration of Acromegaly:*<br>mean 9.1 years	Dose / day<br>Mean 16.5 [SD 7.7]<br>94% patients<br>Range 10 to 30	baseline vs 6, 12 , 24 months PEG	Maximum 24 months	Serum IGF-1<br>Adverse events (injection site reaction, elevated liver enzymes in serum, headache, increase in pituitary tumour volume.
Parkinson 2007[12]<br><br>(Germany, Netherlands, Sweden, UK, and US)<br><br>Many previous participants in Trainer 2000	Multi centre,<br><br>retrospective uncontrolled before and after design<br><br>29 of 147 not evaluable	Diagnosis of acromegaly<br><br>Serum IGF-1 at least 1.3 x the upper limit age-adjusted normal range<br><br>SSA and DopA washout 2 and 5 weeks	Receiving LASSA.	147	Surgery:<br>unclear / not reported<br><br>Radiotherapy<br>58%% of 118<br><br>SSA unclear / not reported<br>DopA unclear / not reported	*Age: yrs*<br>median 44, range 20 - 78.7<br><br>*Sex M/F*<br>58% / 42%<br><br>*Duration of Acromegaly:*<br>mean 9.1 years	Day 1: an 80 mg loading dose, then from 10 mg / day titrated every 8 wks by 5 mg / day as necessary to provide IGF-1 normality.<br><br>Mean dose duration 12 months [SD 7]	Dose PEG required to normalise IGF-1 correlation with Rx, sex, & baseline GH & IGF-1		
